# Adoption value of support vector machine algorithm-based computed tomography imaging in the diagnosis of secondary pulmonary fungal infections in patients with malignant hematological disorders

**DOI:** 10.1515/biol-2022-0765

**Published:** 2023-12-13

**Authors:** Lieguang Chen, Pisheng Zhang, Lixia Shen, Huiling Zhu, Yi Wang, Kaihong Xu, Shanhao Tang, Yongcheng Sun, Xiao Yan, Binbin Lai, Guifang Ouyang

**Affiliations:** Department of Hematology, Ningbo First Hospital, Ningbo, 315010, Zhejiang, China; Department of Hematology, The Affiliated People’s Hospital of Ningbo University, Ningbo, 315040, Zhejiang, China

**Keywords:** computed tomography images, hematological malignancies, support vector machine, pulmonary fungal infection

## Abstract

This study aimed to assess the feasibility of diagnosing secondary pulmonary fungal infections (PFIs) in patients with hematological malignancies (HM) using computerized tomography (CT) imaging and a support vector machine (SVM) algorithm. A total of 100 patients with HM complicated by secondary PFI underwent CT scans, and they were included in the training group. Concurrently, 80 patients with the same underlying disease who were treated at our institution were included in the test group. The types of pathogens among different PFI patients and the CT imaging features were compared. Radiomic features were extracted from the CT imaging data of patients, and a diagnostic SVM model was constructed by integrating these features with clinical characteristics. *Aspergillus* was the most common pathogen responsible for PFIs, followed by *Candida*, *Pneumocystis jirovecii*, *Mucor*, and *Cryptococcus*, in descending order of occurrence. Patients typically exhibited bilateral diffuse lung lesions. Within the SVM algorithm model, six radiomic features, namely the square root of the inverse covariance of the gray-level co-occurrence matrix (square root IV), the square root of the inverse covariance of the gray-level co-occurrence matrix, and small dependency low gray-level emphasis, significantly influenced the diagnosis of secondary PFIs in patients with HM. The area under the curve values for the training and test sets were 0.902 and 0.891, respectively. Therefore, CT images based on the SVM algorithm demonstrated robust predictive capability in diagnosing secondary PFIs in conjunction with HM.

## 1 Introduction

Hematological diseases, also known as blood system diseases, refer to diseases that originate in or mainly involve the blood and hematopoietic organs and can be found in people of different ages [1]. The common diseases include anemia, bleeding, and fever [2], and their causes include genetic defects and drug damage. The main types are iron-deficiency anemia, aplastic anemia, leukemia, and immune thrombocytopenic purpura [3,4]. Early diagnosis and treatment of this disease are crucial, and therefore improving awareness of hematological diseases is crucial [5–7].

Pulmonary fungal infections (PFI) involve fungal inflammation or related lesions in the lungs and bronchi. Symptoms of PFI can be mild or severe, depending on the pathogen and host status [8,9,10]. Blood diseases can lead to weakened immunity and abnormal blood count in patients, thereby increasing the risk of PFI, and causes patients to experience symptoms such as fever, cough, drowsiness, limb weakness, and loss of appetite [11]. Clinical impact assessment of PFI commonly involves chest X-ray examination [12]. Computed tomography (CT) is the most common X-ray imaging technology. With shadows and halo signs visible in the early stages of infection, 10–15 days after the onset, liquefaction, and necrosis of the pulmonary consolidation region are shown in CT, which has a good diagnostic performance [13].

Currently, radiomics can transform images into digital features through machine fitting. In recent years, artificial intelligence algorithms have made significant progress in the field of medical image diagnostics and have been widely applied to the diagnosis and assessment of pulmonary diseases [14–16]. Secondary PFIs in hematological malignancies (HM) can significantly impact patients’ quality of life and prognosis. However, traditional radiological methodologies have certain limitations in accurately diagnosing fungal infections in the lungs. Definitive determination often necessitates further sampling and examination by medical professionals. In recent years, the application of machine algorithms for assisting medical diagnosis has gained widespread acceptance, and the utilization of artificial intelligence-based CT imaging techniques has shown tremendous potential. This research primarily investigated the utility of AI-based CT imaging in the diagnosis of secondary PFIs in patients with HM. The support vector machine (SVM) algorithm has been widely applied in medical imaging, as indicated by previous studies [17,18]. In the context of diagnosing secondary PFIs in HM, SVM demonstrates the capability for precise localization of lesions. Through large-scale sample training, SVM algorithm models achieve higher diagnostic accuracy, effectively distinguishing trained disease images from those of other conditions. This aids in facilitating precise treatment decisions [19]. Furthermore, CT imaging based on the SVM algorithm can expedite the diagnostic process within a relatively short time frame. Secondary PFIs in the context of HM represent a potentially dangerous and severe complication. Leveraging CT imaging technology based on the SVM algorithm offers distinct advantages, particularly in the early detection and treatment of PFIs. This not only enhances the efficiency of healthcare professionals but also ensures patients receive more timely treatment, thus providing robust support for clinical decision-making.

In this study, CT examinations were conducted on a cohort of 100 patients suffering from HM complicated by secondary PFI. Utilizing CT images, an analysis of the pathogens and radiological features observed in different PFI patients was conducted. Subsequently, features from the CT images were extracted using the SVM algorithm. This algorithm was then trained on data from the training set of patients to enhance its diagnostic accuracy. The ultimate goal of this study was to provide valuable insights for the clinical diagnosis and treatment of secondary PFIs in patients with HM. Therefore, this research holds significant application value and broader implications in the field of biology.

## 2 Literature review

Godoy et al. [20] highlighted the susceptibility of patients with HM and hematopoietic stem cell transplant (HSCT) recipients to fungal pulmonary infections. The most common pathogens associated with these infections include *Aspergillus*, *Mucor*, *Rhizopus*, and *Candida* species. Due to the immunocompromised state or immunodeficiency in these patients, fungal pneumonia exhibits a significantly elevated incidence rate. In the research by Lewis et al. [21] focusing on the diagnosis of fungal pulmonary infections in patients with HM, it was emphasized that high-resolution chest CT remains the preferred diagnostic imaging modality for invasive fungal diseases. CT imaging can reveal features such as nodules with or without ground-glass opacities, masses, consolidations, wedge-shaped infarctions, and pleural effusions. Furthermore, with the development of fungal-specific antibody imaging tracers, CT has become an indispensable tool in the diagnosis of this condition. Kunihiro et al. [22] conducted CT examinations on 128 patients with pulmonary infections associated with HM. The infection types among the patients included bacterial pneumonia (37 cases in non-HSCT cases and 14 cases in HSCT cases), Pneumocystis pneumonia (PCP) (29 cases in non-HSCT cases and 11 cases in HSCT cases), and fungal infections other than PCP (20 cases in non-HSCT cases and 17 cases in HSCT cases). After evaluating the CT scans of the patients, the doctors conducted comparisons using chi-square tests and multivariate logistic regression analysis. They observed that CT imaging could distinctly depict features such as centrilobular nodules, nodules with a halo sign, and cystic lesions with air crescent signs in the patients. Furthermore, based on pathogen testing, it was found that patients with *Aspergillus* infections exhibited early CT findings of nodules with a halo sign, while in later stages, they displayed cystic lesions with air crescent signs. Patients with *Cryptococcus* infections exhibited CT imaging features characterized by nodules and cavities, while those with *Candida* infections presented with centrilobular nodules or randomly distributed small nodules. Hałaburda-Rola et al. [23] conducted CT examinations on 35 neutropenic patients with invasive pulmonary aspergillosis who had symptoms such as fever, dyspnea, or nonproductive cough occurring within 72 h. They applied the *European Organization for Research and Treatment of Cancer/Mycoses Study Group* (EORTC/MSG) criteria and found that CT’s diagnostic value increased by 11.4%. Martinez et al. [24] trained and tested various machine learning systems on 264 cases of invasive fungal diseases and 289 control patients. The results indicated that the SVM algorithm model exhibited a high recall rate at the patient level, reaching 95 and 100%, respectively. Qian et al. [25] included 40 patients with pulmonary aspergillosis, divided into a training group (*n* = 28) and a testing group (*n* = 12). They performed CT imaging, extracted radiomic features, and established a diagnostic model based on the SVM algorithm. The area under the curve (AUC) values for the training and testing sets were 0.896 and 0.886, respectively. These findings highlight the SVM algorithm’s remarkably high accuracy in diagnosing pulmonary aspergillosis. In summary, SVM algorithm models exhibit the highest sensitivity in analyzing CT images and can diagnose specific fungal infections based on radiomic features, thereby enhancing diagnostic efficiency.

## 3 Materials and methods

### 3.1 Research objects

One hundred patients with HM complicated by secondary PFI in the hospital from March 10, 2020, to February 10, 2022, were selected as the research objects, serving as training sets. Sixty-eight males and 32 females were included, with an age range of 10–65 years old. The patients in the test set were 80 patients with HM complicated with secondary PFI admitted to our hospital during the same period. Among them, there were 51 males and 29 females, with an age range of 12–70 years. This research had been approved by the ethics committee of the hospital, and the family members of the patients knew about the research and signed the informed consent.

### 3.2 CT imaging examination

The patients were examined by dual-source CT (Manufacturer: Siemens, Germany, Model: SOMATOM Drive) scanner. The patient was in a supine position with the arms raised above the head, and the head went ahead through the instrument. Scanning was from the superior aperture of the thorax to the mid-abdomen. Scanning parameters included a tube voltage of 120 kV, a tube current of 150 mA, a slice spacing of 5.5 mm, and a slice thickness of 5.5 mm. The obtained CT images were sent to the workstation for processing, and the images were read and diagnosed by two senior physicians together. The features of CT images were analyzed.

### 3.3 Image segmentation

**Figure 1:**
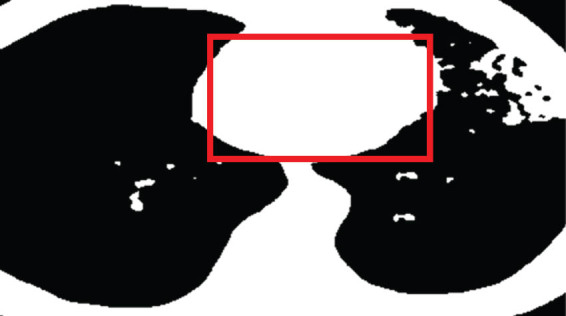
Patient CT image (Red box indicates ROI). The patient was a 55-year-old male with intermittent cough and wheezing for 5 years, aggravating phlegm with blood and high fever for 11 days. Ten days before admission, the patient had no obvious cause of phlegm and blood, and pain in the left chest area (initial examination on admission).

At the critical level of image lesion characterization, the image window width and window level were set to 400 Hounsfield Units (Hu) and 50 Hu, respectively. Subsequently, employing ITK-SNAP 3.8 software, the diagnostic region of interest (ROI) was delineated along the contours of the lesion. The outlined ROI (Figure 1) was then blindly reviewed and outlined by a radiologist from the hospital’s radiology department. The maximum slice image of the focused-enhanced CT along the transverse axis was imported, and corresponding ROI images were drawn into the A.K. software for feature extraction. A total of 24 features were extracted from the axial images of the maximum slice of each lesion, encompassing morphological features, first-order histogram features, second-order histogram features, and higher-order features.

### 3.4 Feature extraction

The research cohort comprised 100 patients with malignant hematological disorders complicated by secondary PFI (research group) and 80 patients with malignant hematological disorders as the control group, aimed at mitigating the influence of dimensionality and value range disparities. Initial feature selection in the training set was conducted using the minimum redundancy maximum relevance algorithm, which eliminated redundant and irrelevant features while retaining those with the highest predictive efficiency:

\[\text{mRMR}=\text{argmax}{f}_{i}\in X\left[I(c;{f}_{i})-1| S| \sum {f}_{j}\in SI({f}_{i};{f}_{j})\right].]\]

The original data were transformed into linear data and mapped between 0 and 1, inclusive:

\[\text{NMIFS}=\text{argmax}\hspace{.25em}{f}_{i}\in X\left[I({f}_{i}\text{;}c)-1| S| \sum {f}_{j}\in SI({f}_{i}\text{;}{f}_{j})\text{min}(H({f}_{i}),H({f}_{j}))\right].]\]

Subsequently, the least absolute shrinkage and selection operator algorithm was employed for further feature selection, and the objective function of the prediction model was presented as follows:

\[\sum {n}_{i}=1\left(\phantom{\rule[-0.75em]{}{0ex}}{y}_{i}-\sum {p}_{j}=1{x}_{ij}{\beta }_{\text{j}}\right)2+\lambda\sum {p}_{j}=1| {\beta }_{j}| =\text{RSS}+\lambda \sum {p}_{j}=1| {\beta }_{j}| .]\]

### 3.5 Establishment of the SVM model

**Figure 2:**
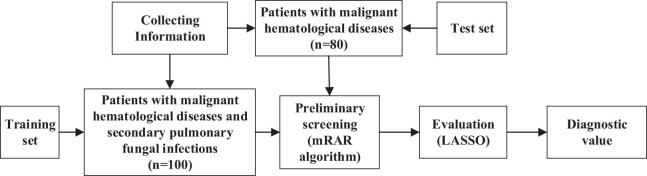
Establishment of SVM algorithm for predicting secondary PFI in malignant hematological disorders.

The SVM model was constructed by integrating clinical factors with CT image features. A non-linear mapping function (Kernel function) was employed to transform the data into a high-dimensional feature space, enhancing the potential linear separability of data within this space. Subsequently, an optimal hyperplane was determined within the feature space, effectively delineating distinct categories of samples. Support vectors, representing the samples closest to the hyperplane, were utilized to compute the distance between support vectors and the hyperplane. This computation facilitated the establishment of decision boundaries and the basis for discriminating new samples. The effectiveness of the models for both the research and control groups was validated. The model is illustrated in Figure 2.

### 3.6 Diagnostic criteria

According to the *Diagnostic Criteria and Therapeutic Principle of Invasive Fungal Infection in Hematological Diseases or Malignant Tumors* [26], the diagnosis of PFI was made. The diagnosis was divided into confirmed, clinically confirmed, probable, and undetermined. Histopathological examination was conducted with the specimens taken from patients included, from which molds were shown with hyphae, yeasts showed yeast cells or hyphae, and sporozoites showed sporozoite cysts or trophozoites.

### 3.7 Observation indicators

The basic data (age, gender, and length of hospital stay) of the patients were collected. The disease types of HM were counted, including acute lymphoblastic leukemia (ALL), myelodysplastic syndrome (MDS), non-Hodgkin’s lymphoma (NHL), and multiple myeloma (MM). The strain categories and their proportions (*Aspergillus*, *Mucor*, *Candida albicans*, *Cryptococcus,* and *Pneumocystis carinii*) were also counted. The CT image features of the patients were statistically analyzed based on the type of fungus infecting the patients. These features included lesion characteristics, diffuse lung lesions, lesion extent, lesion morphology, air crescent sign, and nodule size. Lesion characteristics encompassed multiple lesions and single lesions, diffuse lung lesions included unilateral lung lesions and bilateral lung lesions, and lesion extent covered subpleural lesions, perihilar pulmonary lesions, and other lung areas. Lesion morphology included single form and single form. The air crescent sign represented the formation of a crescent-shaped lucency between spherical lesions or cavities within the lungs and the cavity wall. Nodule size was categorized into patients with nodules measuring ≤3 and >3 cm.

### 3.8 Statistical methods

SPSS19.0 was applied for data processing in this study. The measurement data and the enumeration data were expressed as mean ± standard deviation ( \[\bar{x}]\] ± *s*) and percentage (%), respectively. Pairwise comparisons were made using one-way analysis of variance. The SVM model was employed for diagnostic purposes and assessed the diagnostic efficiency of the model using the receiver operating characteristic (ROC) curve and the AUC. The difference was considered statistically significance at *P* < 0.05.


**Inclusion criteria:** The patients had complete clinical data, normal intelligence, and barrier-free communication, as well as a length of hospital stay for more than 48 h.
**Exclusion criteria:** Patients with psychiatric diseases, those who dropped out of the research due to personal reasons, those who had poor CT image quality, and those with difficulty in listening, writing, and normal communication were excluded.
**Informed consent:** Informed consent has been obtained from all individuals included in this study.
**Ethical approval:** The research related to human use has complied with all the relevant national regulations, and institutional policies in accordance with the tenets of the Helsinki Declaration, and has been approved by the ethics committee of the hospital.

## 4 Results

### 4.1 Basic data of patients with HM

**Figure 3:**
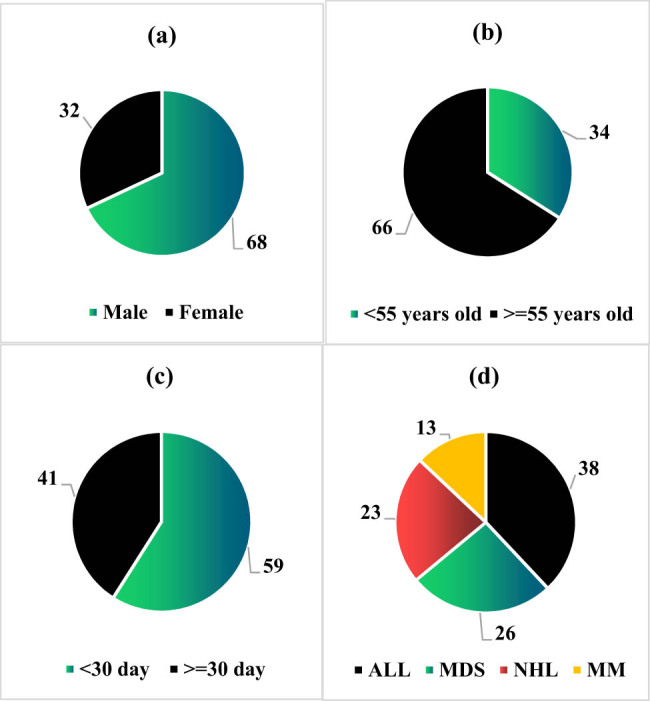
Basic information of patients with malignant hematological tumors in the training set: (a) gender, (b) age, (c) length of hospital stay, and (d) disease types.

Among the 100 patients in the training set with malignant hematological diseases complicated by secondary PFI, 68 were male, and 32 were female. Sixty-six patients were ≥55 years old and 34 patients were less than 55 years old. There were 41 patients with hospital stay ≥30 days, while 59 patients with ≤30 days. For the disease types, 38, 26, 23, and 13 cases had ALL, MDS, NHL, and MM, respectively (Figure 3).

**Figure 4:**
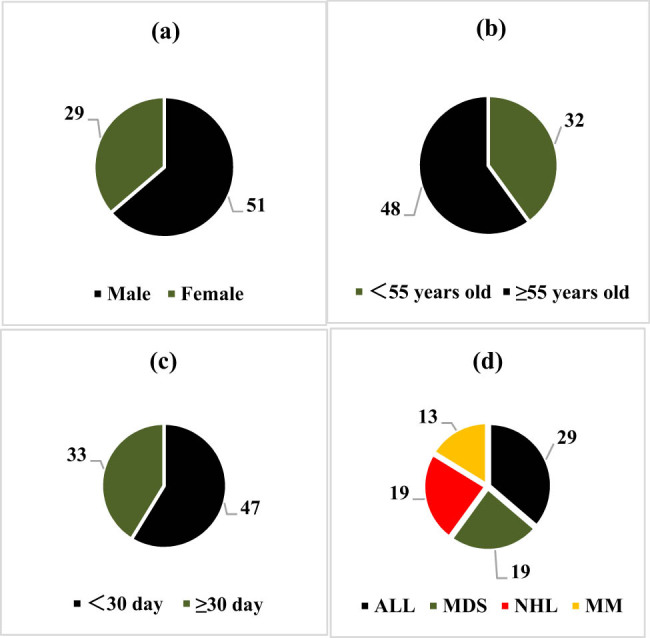
Basic information of patients with malignant hematological tumors in the test set: (a) gender, (b) age, (c) length of hospital stay, and (d) disease type.

In the test set of 80 patients with malignant hematological diseases, there were 51 males and 29 females. Among these, 48 patients were aged 55 years or older, while 32 were younger than 55 years. Additionally, there were 33 patients with hospital stays of 30 days or more and 47 patients with stays of less than 30 days. Regarding disease types, there were 29 patients with ALL, 19 with MDS, 19 with NHL, and 13 with MM (Figure 4).

The analysis of data pertaining to patient characteristics, including gender, age, and disease type, in both the training set and the test set revealed no significant statistical differences (*P* > 0.05).

### 4.2 Strain categories in patients

**Figure 5:**
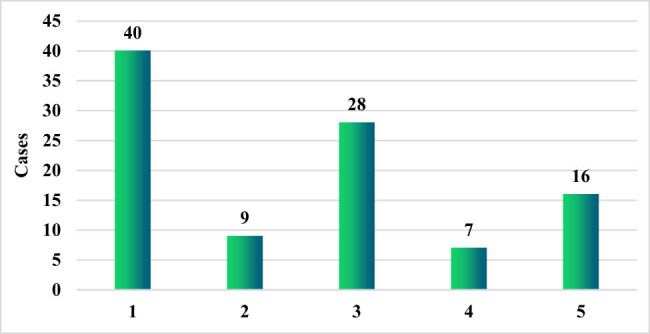
Strain categories in patients. 1–5 represent *Aspergillus, Mucor, Candida albicans, Cryptococcus,* and *Pneumocystis carinii,* respectively.

As shown in Figure 5, among the 100 patients with HM complicated by secondary PFI, the most common pathogen was *Aspergillus* found in 40 cases (40%). It was followed by *Candida albicans* in 28 cases (28%) and then *Pneumocystis carinii* found in 16 cases (16%). The fewest pathogens were *Mucor and Cryptococcus*, which were found in 9 cases (9%) and 7 cases (7%), respectively.

### 4.3 CT features of different PFIs

**Figure 6:**
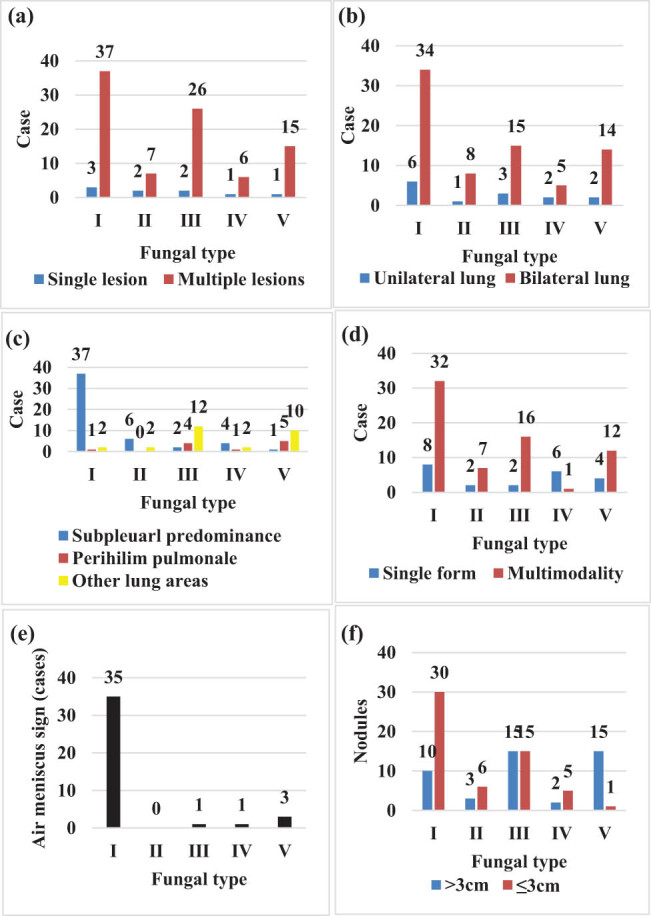
CT features of different PFIs. Ⅰ–Ⅴ represent *Aspergillus, Mucor, Candida albicans, Cryptococcus,* and *Pneumocystis carinii*, respectively: (a) lesion condition, (b) diffuse lung lesions, (c) extent of lesions, (d) morphology of lesions; (e) air meniscus sign, and (f) nodule size.

As shown in Figure 6, among patients with fungal infections such as *Aspergillus, Mucor, Candida, Cryptococcus, and Pneumocystis*, when observing the lesion characteristics, multiple lesions were most common in patients with *Aspergillus* infections. Regarding lesion characteristics, patients with diffuse lung lesions had a significantly higher incidence of bilateral lung infections compared to those with unilateral lung infections. Among these, patients with bilateral lung infections in *Aspergillus* were the most common, followed by *Candida, Pneumocystis, Mucor*, and *Cryptococcus*. Furthermore, patients with *Aspergillus, Mucor, and Cryptococcus* infections mainly had lesions below the pleura, while those with *Candida* and *Pneumocystis* infections predominantly had lesions in other lung regions. In terms of lesion morphology, patients with polymorphic infections were significantly more common than those with a single form among those with *Aspergillus, Mucor, Candida, Cryptococcus,* and *Pneumocystis* fungal infections. When observing patients with air crescent sign, irregular cavities, pleural adhesions, it was found that patients with *Aspergillus* infections had the highest number of patients with air crescent sign, followed by *Pneumocystis, Cryptococcus, Candida,* and *Mucor* infections. Looking at the size of nodules in different infection patients, it was found that patients with *Aspergillus, Mucor,* and *Cryptococcus* infections had significantly more patients with nodules smaller than 3 cm than those with nodules larger than or equal to 3 cm. On the other hand, patients with *Mucor* and *Pneumocystis* infections had significantly fewer patients with nodules smaller than 3 cm compared to those with nodules larger than or equal to 3 cm.

### 4.4 Extraction of image features

**Figure 7:**
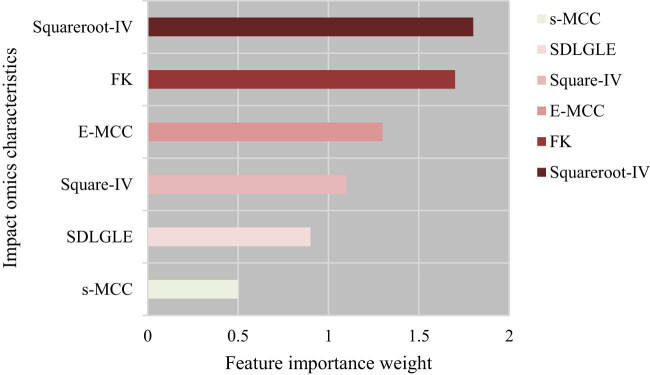
Relative importance ranking of feature data in SVM algorithm.

A total of 24 features were selected from the CT images, and after processing, the six most influential radiomics features were extracted. These features are Squarerooted Grey-Level Co-occurrence Matrix Inverse Variance (Squareroot-IV), Squared Grey-Level Co-occurrence Matrix Inverse Variance (Square-IV), Exponential Maximum Correlation Coefficient (E-MCC), Square-rooted Maximum Correlation Coefficient (s-MCC), First-order Kurtosis (FK), and Small Dependence Low Grey-Level Emphasis (SDLGLE). As shown in Figure 7, the relative importance ranking of these six radiomics features from highest to lowest was Squareroot-IV, FK, E-MCC, Square-IV, SDLGLE, and s-MCC.

### 4.5 Prediction efficiency of the SVM model

**Figure 8:**
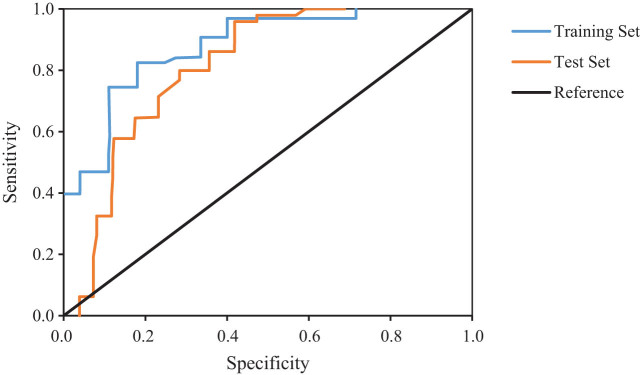
ROC curve of the SVM model.

The diagnostic model consisted of clinical data for malignant hematological disorders complicated by secondary PFI. CT images showed ground-glass opacities in the upper lobe of the left lung, with surrounding halo signs; nodular shadows within the lungs were enlarged and exhibited consolidation, with nodules along the walls appearing in the left upper lobe; diffuse small patchy shadows were observed in the right lung, accompanied by irregularly shaped air crescent signs in the periphery. Six radiomics features were extracted (Figure 8). In the training set, the AUC of the SVM model was 0.902 (95% CI: 0.803–0.924). In the testing set, the model achieved an AUC of 0.891 (95% CI: 0.793–0.901).

### 4.6 Imaging data

**Figure 9:**
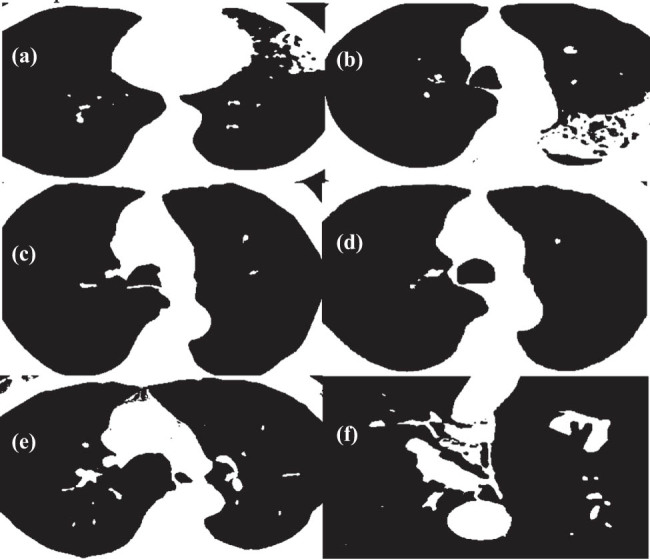
A 55-year-old male patient with intermittent cough and asthma for 5 years, aggravated with bloody sputum, and high fever for 11 days. Ten days before admission, the patient developed bloody phlegm without obvious inducements and had pain in the left anterior thoracic region. (a) and (b) Initial CT images at admission; (b) and (c) CT images during the treatment; and (d)–(f) re-examined CT images 2 months after the treatment.

Figure 9 displays the CT data of a male patient, and the sputum fungal culture of *Aspergillus flavus* was made. Chest CT (Figure 9a and b) showed a patchy ground-glass opacity in the upper lobe of the left lung with a surrounding halo sign. First, the patient was given anti-infective treatment such as cefoperazone, imipenem, and vancomycin combined with voriconazole, but the fever symptoms did not improve obviously. CT examination (Figure 9c and d) was performed again, from which the nodules in the lungs were larger than before, accompanied by consolidation, and wall nodules appeared in the left upper lobe. The patient was then treated with posaconazole, glucocorticoid, and other treatments. After 2 months, the CT re-examination (Figure 9e and f) showed that the left-lung infiltration shadow disappeared, but there were residual linear and patch shadows.

**Figure 10:**
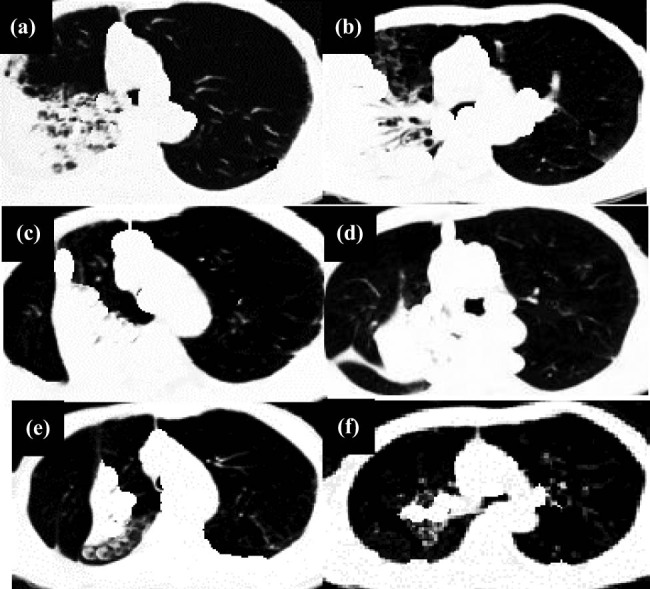
Another 55-year-old male patient with repeated cough for more than 2 years, was admitted to the hospital for recurrent chest pain for 3 days. Physical examination showed a small number of moist rales in the right upper lung. (a) and (b) initial CT images at admission, (b) and (c) initial CT images during the treatment, and (d)–(f) initial CT images 2 months after the treatment.

Figure 10 presents the CT data of another male patient, and the washing fluid culture was conducted with *Candida albicans*. Chest CT (Figure 10a and b) revealed diffuse small patchy shadows in the right lung with an air meniscus sign and irregular borders. First, the patient was treated with voriconazole and caspofungin. After 1 month, an examination was given. The patient was still coughing, and CT (Figure 10c and d) showed that the changes were not notable in the consolidation shadow of the hilum of the right lung, and the patch shadows were not reduced much. After that, amphotericin B and glucocorticoid therapy were given. The CT scan after 2 months (Figure 10e and f) suggested that the consolidation shadow of the right hilum disappeared. The patchy shadows in the lungs shrank, and cavities appeared in the patch in the lateral segment of the right middle lobe.

### 4.7 Prognosis of patients with different PFIs

**Figure 11:**
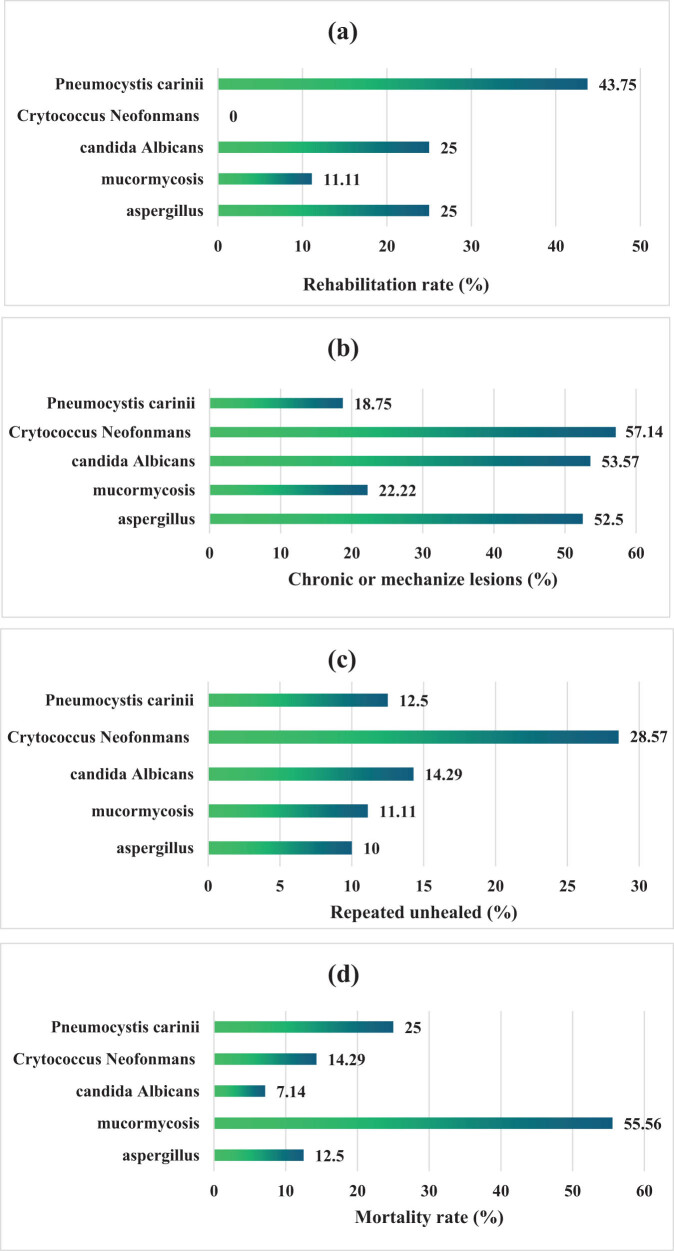
Prognosis of patients with different PFIs. (a)–(d) Recovery rate, chronic or organized lesions, unhealed with recurrent disease, and mortality, respectively.

Figure 11 displays the prognosis of patients with different PFIs. Among patients with *Aspergillus*-induced PFI, 10 cases (25%) recovered, 21 cases (52.5%) had chronic or organized lesions, 4 cases (10%) suffered from the recurrent disease without recovery, and 5 cases (12.5%) died. Among patients with PFI induced by *Mucor*, 1 case (11.11%), 2 cases (22.22%), 1 case (11.11%), and 1 case (11.11%) recovered had chronic or organized lesions, unrecovered with recurrent disease, and died, respectively. Among patients with PFI of *Candida albicans*, 7 (25%), 15 (53.57%), 4 (14.29%), and 2 (7.14%) cases had the above consequences, respectively. Among patients with *Cryptococcus*-induced PFI, 0 cases (0%), 4 cases (57.14%), 2 cases (28.57%), and 1 case (14.29%) encountered the above, respectively. Among patients with PFI of *Pneumocystis carinii*, 7 cases (43.75%) recovered,3 cases (18.75%) had chronic or organized lesions, 2 cases (12.5%) had the disease recurrently, and 4 cases (25%) died. Patients with *Mucor*-induced PFI had the highest mortality, followed by *Pneumocystis carinii*. Patients with PFI of *Pneumocystis carinii* had the highest recovery rate.

## 5 Discussion

Pulmonary infection is the most common symptom of infection, accounting for about 40% of all infections. With the improvement of infection diagnosis and treatment capabilities and the use of new antibiotics in recent years, great progress has been made in the control and management of pulmonary infection in clinical practice. Most patients including those with bacterial infections and fungal infections can be cured [27–29]. Fungal infections have a high incidence in patients with hematological diseases, and some patients have poor prognosis, which affects the course of treatment, treatment costs, and mortality of the underlying diseases to a certain extent. Therefore, it is very necessary to understand the features of different fungal infections in patients with HM [30]. In this research, the results indicated that *Aspergillus* and *Candida albicans* were the major pathogenic bacteria in HM complicated by PFI [31].

CT imaging has significant clinical utility in patients with secondary PFI complicating HM. Due to their compromised immune function, patients with HM are susceptible to bacterial, viral, and fungal infections, with PFIs being a common and severe complication [29,32,33]. CT imaging provides detailed information about pulmonary structures, aiding in the early detection and assessment of the extent of PFI, thereby guiding treatment strategies. In patients with secondary PFI complicating HM, CT imaging provides following valuable insights: (1) Location and distribution of pulmonary lesions: CT can clearly depict the location and distribution of pulmonary infection foci, including unilateral or bilateral involvement, and localized or diffuse distribution, assisting in the determination of the extent and severity of the infection. (2) Morphology and density of lesions: CT can delineate the morphological features of infection foci, such as nodules, cavities, and consolidation, and evaluate lesion density. Different pathogens may cause infections with distinct morphological and density characteristics; thus, CT imaging can offer clues regarding the type of pathogen involved. (3) Signs of concurrent complications: patients with secondary PFI complicating HM often present with complications such as pleural effusion, air bronchogram signs, and pleural effusion. CT aids in detecting the presence and assessing the severity of these complications, providing a basis for a comprehensive evaluation of the patient’s condition. Studies have demonstrated that CT imaging is highly sensitive for the early diagnosis of invasive pulmonary aspergillosis complicating HM. Furthermore, Longhitano et al. [34] have shown that CT imaging can reflect the presence of disseminated infection and is highly sensitive for monitoring infection progression over time. In this research, the CT characteristics of different PFI patients were analyzed, and the results revealed that multiple lesions are most commonly observed in patients with *Aspergillus* PFI. The diffuse lung lesions were observed in bilateral lungs, and the lesions were mainly subpleural. Air meniscus signs, irregular cavities, and pleural adhesions could be observed, and the nodules were basically less than 3 cm. Multiple lesions were also the most common in patients with *Mucor*-induced PFI. The diffuse lung lesions in bilateral lungs and the mainly subpleural lesions were on display. None of the air meniscus signs was discovered, and the nodules were mostly less than 3 cm. Patients with Cryptococcus-induced PFI were also predominant with multiple lesions, together with bilateral-lung diffuse lesions, no air meniscus sign, irregular cavities, and nodules less than 3 cm. All patients with PFI of *Candida albicans* had multiple lesions, with various morphologies, bilateral-lung diffuse lesions, most lesions in other lung regions, and nodules more than 3 cm. Thus, from the CT image features, it could be concluded that patients with different PFIs were shown differently in terms of lesion condition, lesion extent, lesion morphology, and nodule size. Therefore, CT could be utilized for the assessment of PFIs in patients with hematological diseases. From the prognosis of the patients, the patients with *Mucor*-PFI had the highest mortality (55.56%), followed by 25% of *Pneumocystis carinii*-induced PFI. The patients with *Pneumocystis carinii*-PFI had the highest recovery rate of 43.75%. Ghuman et al. [35] suggested that mucormycosis affecting the intestinal tract had a high mortality, and early identification and intervention could considerably improve the prognosis of patients. The disease should be suspected in immunosuppressed patients as their imaging findings showed unexplained bowel ischemia, infarction, and/or pneumonia without any obvious vascular thrombosis. The results here were similar.

Analysis revealed no significant statistical differences in gender, age, and disease type between the training and testing sets. This suggests a relatively balanced distribution of patient gender, age, and disease type in both the training and testing datasets. The lack of significant statistical differences indicates a similarity in data distribution between the training and testing sets, enhancing the model’s generalization ability in real-world scenarios. In terms of image feature extraction, six optimal radiomics features were extracted from the 24 CT images. These included Squareroot-IV, Square-IV, E-MCC, s-MCC, FK, and SDLGLE. These features are considered to play a significant role in the diagnostic model for malignant hematological disorders complicated by secondary PFI. Squareroot-IV, for example, can be utilized to characterize the complexity of texture in images, while FK reflects peakness information in the grayscale distribution of images [36]. The extraction of these features aids the model in comprehending latent diagnostic information within the images, thereby enhancing predictive accuracy. The evaluation of the SVM model’s predictive performance on the training and testing sets involves measuring its performance through the calculation of the AUC of the ROC curve. The SVM model yielded an AUC of 0.902 (95% CI: 0.803–0.924) on the training set, and an AUC of 0.891 (95% CI: 0.793–0.901) on the testing set. A higher AUC value closer to 1 indicates better model performance. This suggests that the SVM model demonstrates strong predictive capability in diagnosing malignant hematological disorders complicated by secondary PFI. The results indicate that the model can accurately predict whether a patient is affected by PFI based on their CT images and radiomics features, thereby providing a basis for assisting medical professionals in making informed decisions.

## 6 Conclusion

In this research, CT examinations were conducted on a cohort of 100 patients with HM complicated by secondary PFI. The findings revealed that the nature of lesions, their extent, morphology, and nodule sizes varied among patients with different PFIs. Therefore, CT imaging can be effectively utilized for assessing PFIs in hematological disease patients. Furthermore, the SVM model constructed based on extracted radiomic features exhibited strong predictive performance in diagnosing secondary PFIs in patients with HM. These results indicate that SVM algorithm models can be employed for rapid diagnosis when dealing with the diagnosis of secondary PFIs in HM. These findings hold significant clinical relevance for improving the diagnosis of complications in HM. This research had a relatively small sample size, consisting of only 100 patients with HM complicated by secondary PFI. Future research could benefit from expanding the sample size, including a more diverse range of hematological malignancy types, to validate and generalize the applicability of this diagnostic approach. While the SVM algorithm model demonstrated strong predictive capabilities in diagnosing secondary PFI, there is scope for further optimization and improvement of the model performance. Future research efforts may explore the use of other machine learning algorithms or deep learning methods to enhance diagnostic accuracy and stability. Additionally, future studies could continue to investigate the influence of different pathogen types on CT imaging features and develop more precise prognostic assessment models.
